# The probabilities of type I and II error of null of cointegration tests: A Monte Carlo comparison

**DOI:** 10.1371/journal.pone.0259994

**Published:** 2022-01-04

**Authors:** Ahmet Faruk Aysan, Ibrahim Guney, Nicoleta Isac, Asad ul Islam Khan

**Affiliations:** 1 College of Islamic Studies, Hamad Bin Khalifa University, Doha, Qatar; 2 Faculty of Education, Istanbul Sabahattin Zaim University, Istanbul, Turkey; 3 Faculty of Business and Management Sciences, Istanbul Sabahattin Zaim University, Istanbul, Turkey; 4 Faculty of Humanities and Social Sciences, Ibn Haldun University, Istanbul, Turkey; Istanbul Medeniyet University: Istanbul Medeniyet Universitesi, TURKEY

## Abstract

This paper evaluates the performance of eight tests with null hypothesis of cointegration on basis of probabilities of type I and II errors using Monte Carlo simulations. This study uses a variety of 132 different data generations covering three cases of deterministic part and four sample sizes. The three cases of deterministic part considered are: absence of both intercept and linear time trend, presence of only the intercept and presence of both the intercept and linear time trend. It is found that all of tests have either larger or smaller probabilities of type I error and concluded that tests face either problems of over rejection or under rejection, when asymptotic critical values are used. It is also concluded that use of simulated critical values leads to controlled probability of type I error. So, the use of asymptotic critical values may be avoided, and the use of simulated critical values is highly recommended. It is found and concluded that the simple LM test based on KPSS statistic performs better than rest for all specifications of deterministic part and sample sizes.

## 1. Introduction

The concept of cointegration was firstly proposed by [1]. If two or more than two integrated of order one variables possess a long run relationship, then it is termed as existence of cointegration among them. For two variables *X* and *Y*: integrated of order one, if their linear combination: *aX* + *bY* is integrated of order zero, then *X* and *Y* are possessing a long run relationship and they are said to be cointegrated. Note that cointegration analysis is based on the issue that all variables must be I(1), but this may depend on selecting the structural breaks (see, e.g., [2]). Soon after the development of concept of “cointegration”, a huge variety of tests were proposed to test it like [3–5] and many more. Most of these tests proposed were testing the null of no cointegration. These tests have been widely and frequently used in economics and finance to assess the long run relationship between a set of time series. Some of these studies are but not limited to; [6–10]. In their pioneered paper, [11] proposed first test assessing the null of cointegration. After, this pioneer paper, more cointegration tests evaluating the null of cointegration were developed, assuming different underlying data generation ([12,13] and many others).

As all of these proposed tests were based on different underlying assumptions about the cointegrated system and were assuming different data generations, so they were showing different conclusions about the existence of cointegration for the same empirical problem ([14,15]). Therefore, there was a need to assess their performance and to choose winner/winners among the tests which best fits an empirical problem. To fill this vacuum in literature, numerous comparative studies have been published. Most of these studies used Monte Carlo Simulations (MCS) to evaluate and assess the performance of tests. However, there are fewer real data based comparative studies of cointegration tests ([16,17]). MCS have been frequently used for such comparative studies ([18–23]). These studies ([14,15,24–26] and many more), using MCS were assessing and evaluating the performance of tests based on two properties, the size: “the probability of rejection of null hypothesis when actually it is true” and the power: “the probability of rejection of null hypothesis when actually it is false”. A test is regarded and considered better than other, if it has a controlled size around nominal size and has relatively higher power than the others. Most of these comparative studies of cointegration tests were considering a limited number of alternative hypotheses (2 to 4), although the alternative hypothesis can take on infinite values in an alternative space. Two studies i.e. of [14] and [15] were the most extensive ones. They considered different data generating processes and considered more than 8 point alternative hypotheses, trying to cover the whole alternative space. [14] and [15] concluded that there is no significant evidence that one test is superior than others. According to them, if one test is performing better for some subset of alternative hypothesis, then the same test is performing worst in another subset of alternative hypothesis. So, a general conclusion is very difficult to draw.

Except the study of [27], all of comparative studies of cointegration tests were either addressing a selected set of null of no cointegration tests or a selected set of mixture of both kind of tests having opposite null hypothesis. [27] compared 6 tests (null of cointegration) on basis of the same size and power properties. Although, [27] considered different data generating processes and estimated the power curves, however, the conclusions are same as [14] and [15]. We are not able to find any other comparative study of null of cointegration tests.

As these tests are based on different underlying assumptions and for a particular real data set, one does not know that whether the data satisfies the assumptions of the test or not, therefore the selection of the test is a very critical decision. Therefore this study compares the tests on a very basic type of data generations and it is assumed that if a test is performing poor here, it will be doing the same for other data generations. However, it cannot be confirmed that the best performer will remain the best for other data generations. Moreover, majority of the comparative studies of cointegration tests, in the literature, didn’t use size adjusted powers. These studies were comparing the tests on basis of size and power and if the tests have size distortions then for power comparison, these size distortions were not controlled. Moreover, fewer number of alternative hypothesis and data generations were considered to assess the relative performance. The aim of this paper is twofold: comparison of tests on basis of the probability of Type I error, known as size of test using asymptotic critical values or distributions developed by the respective authors, controlling the probability of Type I error around the assumed nominal probability of Type I error (usage of simulated critical values) and comparison of tests based on probability of Type II error when the probability of Type I error is controlled around a nominal level. These probabilities are defined as:

$$\alpha =Prob\left(Type\;I\;Error\right)=Prob\left(Rejecting\;H_{0}|H_{0\;}is\;True\right)$$

$$\beta =Prob\left(Type\;II\;Error\right)=Prob\left(Failing\;to\;Reject\;H_{0}|H_{0\;}is\;False\right)$$

*α* is also known as Size of the test. Whereas, Power of the test is (1 − *β*). The conclusions and recommendations of this study will be beneficial to a large audience: practitioners, statisticians, data scientists and applied researchers, as it will give the guidelines about a better performing test and worst performing test. These classifications of better and worst performance will be based on *α* and *β*. This study will also be helpful to the audience for the selection of type of critical values.

This study is structured as: next section of “Methodology” elaborated the details of tests and the framework followed to assess the performance of these. “Methodology” is followed by “Results and Discussion” section, discussing the results in greater detail and then the last section is “Conclusions and Recommendations”. The “References” are listed at the end.

## 2. Methodology

In this study, eight tests belonging to the class of null of cointegration are compared, the details of these tests are laid out in “Tests to be compared”. The next section “Artificial Data Generation” lists the set of equations used to generate a cointegrated system and the procedure of estimation of *α* (for both asymptotic and simulated critical values is detailed in "Estimation of Empirical *α*". Continuingly, the next section "Estimation of Simulated Critical Values" details out the steps followed to obtain simulated critical values by fixing *α* and the next section "Estimation of *β*" specifies the steps to be followed to estimate *β*, the probability of Type II Error in. MATLAB has been used to carry out the analysis.

### 2.1. Tests to be compared

The eight tests have been compared in the current study and these tests were selected on basis of their frequently use in the economics/econometrics literature. Moreover, these are the pioneer null of cointegration tests. Furthermore these tests have been chosen from the previous studies like [14,15,27] on the basis of their relative performance. The eight tests compared in this study are detailed as:

#### 2.1.1. LM test based on KPSS statistic (LM)

Considering two different kinds of variables say *Z*_*t*_ and *W*_*it*_, ∀ *i* = 1, 2,– – – –, *k* where both of these kinds of variables are integrated of order one i.e. *I*(1). [11] proposed the estimation of Ordinary Least Squares (OLS) regression first, i.e.

$$Z_{t}=\delta \psi_{t}+\sum_{i=1}^{k}\beta_{i}W_{it}+\upsilon_{t}\;\text{t}=1,2,-----,\text{T}$$ (1)

Where *Z*_*t*_ and *W*_*t*_ are dependent and independent variables respectively. *ψ*_*t*_ represents the deterministic part. [11] proposed that that the LM type test introduced by [28] for testing stationarity of time series, can be used for testing the null of cointegration. i.e. $LM=T^{-2}\frac{\sum_{j=1}^{T}R_{j}^{2}}{{ƛ}^{2}\left(m\right)}$ where $R_{j}=\sum_{t=1}^{j}{\hat{\upsilon }}_{t}$ and ${ƛ}^{2}\left(m\right)=\frac{{\hat{\upsilon }}_{t}^{\prime }{\hat{\upsilon }}_{t}}{T}$. The ${\hat{\upsilon }}_{t}$ are the residuals estimated from Eq (1). The LM test does not follow any regular distribution, therefore the current study uses the critical values of the LM as provided in [29].

#### 2.1.2. Leybourne and McCabe’s test (LBI)

[11] proposed that the same LM test with a non-parametric modification can also be used for the said purpose as stated by [11]. Actually, they proposed a long run variance as compared to the contemporaneous variance, i.e.

$$LBI=T^{-2}\frac{\sum_{j=1}^{T}R_{j}^{2}}{{ƛ}^{2}\left(m\right)}$$

where $R_{j}=\sum_{t=1}^{j}{\hat{\upsilon }}_{t}$ and ${ƛ}^{2}\left(m\right)=T^{-1}\sum_{t=1}^{T}{\hat{\upsilon }}_{t}^{2}+2T^{-1}\sum_{s=1}^{m}\sum_{t=s+1}^{T}{\hat{\upsilon }}_{t}{\hat{\upsilon }}_{t-s}$. Again ${\hat{\upsilon }}_{t}$ are the residuals estimated from Eq (1). The lag truncation parameter *m* is very vital and plays a crucial role in empirical studies. According to [15], the size and power of the test depends on it. The current study is considering *l* = 4, as recommended by [15] i.e. at this value, size of test is controlled around the nominal size and also the test has reasonable powers. Same as the LM test, the LBI does not follow any standard statistical distribution, therefore this study is using the critical values for LBI test as provided [29].

#### 2.1.3. Shin’s C test (Sc)

[12] proposed two modifications to the same LM type test. First [12] proposed to use the Dynamic OLS (DOLS) regression and secondly [12] proposed to use a weighting kernel in estimating the long run variance. The DOLS regression is

$$Z_{t}=\delta \psi_{t}+\gamma W_{t}+\sum_{i=-r}^{r}\pi_{i}\Delta W_{t-i}+\upsilon_{t}\;\text{t}=1,2,-----,\text{T}$$ (2)

Where *Z*_*t*_ and *W*_*t*_ are dependent and independent variables matrices of order *T* × 1 and *T* × *m* respectively. The same LM type test, in this case named as *C* is

$$C=T^{-2}\frac{\sum_{j=1}^{T}R_{j}^{2}}{{ƛ}^{2}\left(m\right)}$$

where $R_{j}=\sum_{t=1}^{j}{\hat{\upsilon }}_{t}$ and ${ƛ}^{2}\left(m\right)=T^{-1}\sum_{t=1}^{T}{\hat{\upsilon }}_{t}^{2}+2T^{-1}\sum_{s=1}^{m}\left(1-ƛ{\left(m+1\right)}^{-1}\right)\sum_{t=s+1}^{T}{\hat{\upsilon }}_{t}{\hat{\upsilon }}_{t-s}$

Again, same as LM and LBI, ${\hat{\upsilon }}_{t}$ are the residuals estimated from Eq (2) using OLS. The selection of lag truncation parameter *m* is again vital for the performance of test as it has been already discussed in section 2.1.2. However, the current study is considering *m* = 4 as proposed by [15]. Similarly [12] recommended *r* = 5, so the current study is using *r* = 5. The critical values for the test are used from [12].

#### 2.1.4. McCabe—Leybourne -Shin test (Ls)

For the same LM type test, a different estimation methodology and process was proposed by [29]. [29] proposed the use of Maximum Likelihood Estimation (MLE) in place of OLS. They proposed that first the residuals from DOLS regression may be estimated i.e. ${\hat{\upsilon }}_{t}$ and then the residuals ${\hat{\eta }}_{t}$ may be estimated using MLE from the regression ${\hat{\upsilon }}_{t}=\sum_{i=1}^{p}\tau_{i}\Delta {\hat{\upsilon }}_{t}{}_{-i}+\eta_{t}$. According to them the selection of *p* is on the basis of the minimum Akaike information Criterion (AIC). The modified LM test statistic now named as *Ls*

$$Ls=\frac{{\hat{\eta }}_{t}^{\prime }\Omega {\hat{\eta }}_{t}}{T^{2}S^{2}\left(m\right)}$$

where Ω = *PP*′ for *P* being the lower triangular matrix of ones. Similarly, $S^{2}\left(m\right)=\frac{{\hat{\eta }}_{t}^{\prime }{\hat{\eta }}_{t}}{T}$. The current study is considering 4 as the maximum value of *p* and the critical values are used from [29].

#### 2.1.5. Hausman H_1_ and H_2_ tests (H_1_ and H_2_)

A test statistic comparing two estimators was proposed by [13]. According to [13], under the null of cointegration both of the estimators are consistent. However, under the alternative of no cointegration one estimator is inconsistent. One estimator is of *γ* say ${\hat{\theta }}_{L}$, when Eq (2) is estimated using DOLS estimation and for the second estimator, estimate the regression and obtain

$$\omega_{t}=Z_{t}-\sum_{j=-r}^{r}{\hat{\pi }}_{j}\Delta W_{t-j}$$ (3)

${\hat{\pi }}_{j}$ being the OLS estimates from Eq (2). The following regression is used to estimate the second estimator say ${\hat{\theta }}_{D}$ i.e.


$$\Delta \omega_{t}=\theta_{D}\Delta W_{t}+\varepsilon_{t}$$ (4)

The two estimators ${\hat{\theta }}_{L}$ and ${\hat{\theta }}_{D}$ are used to estimate both of the Hausman type test statistics i.e. $H_{1}={\left({\hat{\theta }}_{L}-{\hat{\theta }}_{D}\right)}^{\prime }{\left({\hat{\psi }}_{D}+{\hat{\psi }}_{L}\right)}^{-1}\left({\hat{\theta }}_{L}-{\hat{\theta }}_{D}\right)$ and $H_{2}={\left({\hat{\theta }}_{L}-{\hat{\theta }}_{D}\right)}^{\prime }{\hat{\psi }}_{D}^{-1}\left({\hat{\theta }}_{L}-{\hat{\theta }}_{D}\right)$. Where ${\hat{\psi }}_{L}$ and ${\hat{\psi }}_{D}$ are the estimated variance covariance matrices of ${\hat{\theta }}_{L}$ and ${\hat{\theta }}_{D}$ respectively. The critical values of the two tests have been taken from [13] in the current study.

#### 2.1.6. Hansen’s *L*_*c*_ test (Lc)

Fully modified estimation method [30] was used by [31] to propose a test of null of cointegration. The fully modified estimation method involves the estimation of Eq (1) by OLS and the OLS residuals are obtained, i.e. ${\hat{\upsilon }}_{t}$. Define another vector of difference as Δ*W*_*it*_ = *μ*_*it*_ for i = 1,2,---------,k. Define the matrix *ζ*_*t*_ as *ζ*_*t*_ = (*υ*_*t*_, *μ*_*t*_′). Estimates a VAR(1) model for *ζ*_*t*_ as *ζ*_*t*_ = Φ*ζ*_*t*−1_ + *υ*_*t*_. Use the estimated residuals ${\hat{\nu }}_{t}$ to find the ${\hat{\Lambda }}_{\nu }=\sum_{l=0}^{T}w\left(l/\hat{M}\right)\frac{1}{T}\sum_{t=l+1}^{T}{\hat{\nu }}_{t-s}{\hat{\nu }}_{t}\prime$ and ${\hat{\Omega }}_{\nu }=\sum_{l=-T}^{T}w\left(l/\hat{M}\right)\frac{1}{T}\sum_{t=l+1}^{T}{\hat{\nu }}_{t-s}{\hat{\nu }}_{t}\prime$. Where $w\left(l/\hat{M}\right)$ being an appropriate weighting scheme. The current study is using the quadratic spectral kernel i.e. $w\left(l/\hat{M}\right)=\frac{25}{12\pi^{2}{\left(l/\overset{⌢}{M}\right)}^{2}}\left\{\frac{\text{sin}\left(6\pi \left(l/\overset{⌢}{M}\right)\right)/5}{\left(6\pi \left(l/\overset{⌢}{M}\right)\right)/5}-\text{cos}\left(\left(6\pi \left(l/\overset{⌢}{M}\right)\right)/5\right)\right\}$. The automatic bandwidth estimator is given as

$$\hat{M}=1.3221{\left\{\left(\hat{\delta }\left(2\right)\right)T\right\}}^{1/5}\;\text{and}\;\hat{\delta }\left(2\right)=\sum_{a=1}^{p}\frac{4{\hat{\rho }}_{a}^{2}{\hat{\sigma }}_{a}^{4}}{{\left(1-{\hat{\rho }}_{a}\right)}^{8}}/\sum_{a=1}^{p}\frac{{\hat{\sigma }}_{a}^{4}}{{\left(1-{\hat{\rho }}_{a}\right)}^{4}}$$ (5)

${\hat{\rho }}_{a}$ and ${\hat{\sigma }}_{a}^{2}$ are the “a^th^” endogenous variable’s estimated AR coefficient and variance of residuals. The estimates ${\hat{\Lambda }}_{\nu }$ and ${\hat{\Omega }}_{\nu }$ are recolored to obtain $\hat{\Omega }={\left(I-\hat{\Phi }\right)}^{-1}{\hat{\Omega }}_{\nu }{\left(I-\hat{\Phi }\prime \right)}^{-1}$ and $\hat{\Lambda }={\left(I-\hat{\Phi }\right)}^{-1}{\hat{\Lambda }}_{\nu }{\left(I-\hat{\Phi }\prime \right)}^{-1}{\left(I-\hat{\Phi }\right)}^{-1}\hat{\Phi }\hat{\Sigma }$. Where $\hat{\Sigma }=\frac{1}{T}\zeta_{t}\zeta_{t}\prime$. The variance covariance matrices i.e. $\hat{\Omega }$ and $\hat{\Lambda }$ are partitioned as $\Omega =\left[\begin{array}{cc} \Omega_{\upsilon \upsilon } & \Omega_{\upsilon \mu }\\ \Omega_{\mu \upsilon } & \Omega_{\mu \mu } \end{array}\right]\;\text{and}\;\Lambda =\left[\begin{array}{cc} \Lambda_{\upsilon \upsilon } & \Lambda_{\upsilon \mu }\\ \Lambda_{\mu \upsilon } & \Lambda_{\mu \mu } \end{array}\right]$. To obtain the Fully Modified OLS (FMOLS) estimator, following are estimated first

$$\Omega_{\upsilon .\mu }=\Omega_{\upsilon \upsilon }-\Omega_{\upsilon \mu }\Omega_{\mu \mu }^{-1}\Omega_{\mu \upsilon }$$ (6)

and $\Lambda_{\mu \upsilon }^{FM}=\Lambda_{\mu \upsilon }-\Lambda_{\mu \mu }\Omega_{\mu \mu }^{-1}\Omega_{\mu \mu }$. Then the FMOLS estimator is ${\hat{\beta }}^{FM}=\left[\sum_{t=1}^{T}\left(Z_{t}^{FM}W_{t}\prime -\left(0\;{\hat{\Lambda }}_{\mu \upsilon }^{FM}{}^{\prime }\right)\right)\right]{\left[\sum_{t=1}^{T}W_{t}W_{t}\prime \right]}^{-1}$, where

$$Z_{t}^{FM}=Z_{t}-{\hat{\Omega }}_{\upsilon \mu }{\hat{\Omega }}_{\mu \mu }^{-1}\Delta W_{t}\;\text{and}\;{\hat{\upsilon }}_{t}^{FM}=Z_{t}^{FM}-{\hat{\beta }}^{FM}W_{t}$$ (7)

are the estimated FMOLS residuals. [31] proposed the *L*_*c*_ statistic as $L_{c}=trace\left[{\left(\sum_{t=1}^{T}W_{t}W_{t}\prime \right)}^{-1}\sum_{t=1}^{T}{\hat{R}}_{t}{\hat{\Omega }}_{\upsilon •\mu }^{-1}{\hat{R}}_{t}\prime \right]$ and ${\hat{R}}_{t}$ is defined as ${\hat{R}}_{t}=\sum_{i=1}^{t}\left(W_{i}{\hat{\upsilon }}_{i}^{FM}-\left[\begin{array}{c} 0\\ {\hat{\Lambda }}_{\mu \upsilon }^{FM} \end{array}\right]\right)$. The critical values of the [31] proposed *L*_*c*_ test are taken from [26] for the current study.

#### 2.1.7. Xiao Fluctuation test of Cointegration (F)

On the basis of fluctuations of the estimated residuals ${\hat{\upsilon }}_{t}$, [32] proposed a test using FMOLS estimation. It is given as $F_{T}=\underset{i=1,\ldots \ldots \ldots ,T}{\text{max}}\frac{i}{\sqrt{\Omega_{\upsilon \cdot \mu }T}}\left|\frac{1}{i}\sum_{t=1}^{i}{\hat{\upsilon }}_{t}^{FM}-\frac{1}{T}\sum_{t=1}^{T}{\hat{\upsilon }}_{t}^{FM}\right|$ where ${\hat{\upsilon }}_{t}^{FM}$ are the estimated FMOLS residuals and are obtained using Eq (7). Similarly, Ω_*υ*·*μ*_ is obtained using Eq (6). The critical values of *F*_*T*_ are taken from [32] in the current study.

The abbreviations used in the current study for all eight tests are listed in Table 1.

**Table 1:** Abbreviations used for cointegration tests.

S. No	Name of Test	Abbreviation
1	LM test	LM
2	Leybourne and McCabe’s LBI test	LBI
3	Shin’s C test	Sc
4	McCabe—Leybourne -Shin test	Ls
5	Hausman H_1_ test	H1
6	Hausman H_2_ test	H2
7	Hansen’s *L*_*c*_ test	Lc
8	Xiao Fluctuation test	F

### 2.2. Artificial Data Generation (ADG)

The set of equations used to generate a cointegrated system under null of cointegration and alternative of no cointegration are a modified version of the model used by [33]. The [33] model has been modified to include deterministic component also. For time series *z*_*t*_ and *w*_*t*_ of length *T*, the set of equations are:

$$Z_{t}=\psi_{t}\varphi \prime +w_{t}+\upsilon_{t},w_{t}=w_{t-1}+\mu_{t}^{w},\upsilon_{t}=\upsilon_{t-1}-\gamma \mu_{t-1}^{z}+\mu_{t}^{z}$$

where $\mu_{t}=\left(\mu_{t}^{z},\mu_{t}^{w}\right)$ provided that *μ*_*t*_ ~ *N* (0, *I*), *I* being an identity matrix of same order as *μ*_*t*_. Under null hypothesis of cointegration and alternative hypothesis of no cointegration *H*_0_: *γ* = 1 vs *H*_*A*_: 0 ≤ *γ* < 1. The current study takes into account ten different point alternate hypotheses, i.e. *γ* = (0, 0.1, 0.2,––, 0.9). *ψ*_*t*_ is the included deterministic component in ADG and it consists of two deterministic parts; one is the intercept and the other is linear time trend. i.e. $\psi_{t}={\left[\begin{array}{ccccc} 1 & 1 & - & - & 1\\ 1 & 2 & - & - & T \end{array}\right]}^{\prime }$. The *φ* is the respective coefficient vector of *ψ*_*t*_. Three different and plausible cases of deterministic component have been considered in the current study and these are:

i The absence of both Intercept and Linear Time Trend (*denoted as D*^*0*^*LT*^*0*^): *φ* = [0 0]
ii The presence of only the Intercept (denoted as *D*^*1*^*LT*^*0*^): *φ* = [1 0]
iii The presence of both the Intercept and Linear Time Trend (*denoted as D*^*1*^*LT*^*1*^): *φ* = [1 1]

The current study is considering ten point alternate hypothesis from the alternate space and from the null space, one point null hypothesis. Moreover, the performances of tests at four different time lengths (T = 30, 60, 120 and 240) covering the small time lengths to large time lengths are assessed. In addition to these, three different and plausible cases of deterministic parts are explored. This all sums up that 132 different ADG processes have been explored to evaluate the performance of tests.

### 2.3. Estimation of empirical *α*

The current study assesses the performance of test based on two *α′s*: one uses the asymptotic critical values and the other uses the simulated critical values. Nonetheless, their estimation procedure is same as:

i Generation of the time series following the ADG for null hypothesis.
ii Estimation of the test statistic of each test.
iii Taking decision about the rejection of null or not: (based on asymptotic or simulated critical value)
iv The repetition of the steps i, ii and iii for *M* time and counting of number of rejections of null.
v Estimation of the *α* as the proportion of rejections of null out of total *M* repeatitions.

### 2.4. Estimation of simulated critical value

The current study uses the frequently used significance level of 0.05 for whole of its empirical analysis. Estimation of simulated critical values have been carried out by following the below mentioned steps:

i Generation of data under the point null hypothesis.
ii Calculation of each test’s test statistic.
iii Repetition of the steps i and ii for fixed number of times say *M*.
iv Recording of all of *M* test statistics in an array say *S*.
v Calculation of the simulated critical value as: 2.5^th^ and 97.5^th^ percentiles of *S*, for two tailed tests, 95^th^ percentile of *S*, for right tailed tests and 5^th^ percentile of *S*, for left tailed tests.

### 2.5. Estimation of *β*

Considering the frequently used significance level of 0.05, the below mentioned steps have been followed to estimate the Probability of Type II Error *β*.

i Generation of data following a point alternative hypothesis.
ii Estimation of the test statistic.
iii Deciding about the rejection of null or not (based on simulated critical value).
iv Repetition of the steps i, ii and iii for a fixed number of times say *M* and counting of number of rejections.
v Calculation of *β* i.e. *β* = 1 –*π* where *π* = *Number of Rejections/M*

## 3. Results and discussion

The empirical probability of Type I Error i.e. *α* is obtained using *M* = 30,000 for each test except the Ls test, as it uses the numerical optimization algorithms to find the maximum likelihood estimates, which take huge amount of time. So, for Ls: *M* = 5,000 is considered suitable. These *α*′*s* are given in Table 2. It is obvious from Table 2 that not a single test has empirical *α* around the considered significance level of 0.05 for all three specifications of deterministic part and four sample sizes. For instance, LM has *α* around 0.05 for all four sample sizes only when deterministic part is *D*^*1*^*LT*^*0*^. The values of empirical *α* around the specified significance level 0.05 have been marked as BOLD in Table 2. It is observed that from out of total 96 different cases, only for 15 cases the empirical *α* is around 0.05. This over rejection problem is due to the use of asymptotic critical values. As we have finite sample sizes, therefore nearly all of the tests exhibit the over rejection problem. The same was also stated by [34].

**Table 2:** Probabilities of type I error "*α*" using asymptotic critical values.

Tests	D^0^LT^0^				D^1^LT^0^				D^1^LT^1^			
	Time Length *T*				Time Length *T*				Time Length *T*			
	30	60	120	240	30	60	120	240	30	60	120	240
**LM**	0.2271	0.2256	0.2194	0.2232	**0.0589**	**0.0534**	**0.0575**	**0.0533**	0.0002	0.0007	0.0004	0.0002
**H1**	0.0076	0.0080	0.0065	0.0049	0.0023	0.0046	0.0051	0.0061	0.0033	0.0114	0.0148	0.0170
**H2**	0.0111	0.0075	0.0064	0.0056	0.0055	0.0060	0.0063	0.0064	0.0193	0.0161	0.0166	0.0177
**Sc**	0.0048	0.0186	0.0238	0.0252	**0.0638**	**0.0537**	**0.0498**	**0.0528**	0.2034	**0.0573**	**0.0521**	**0.0501**
**LBI**	0.3407	0.2840	0.2464	0.2384	0.2134	0.1299	0.0797	**0.0594**	0.1068	**0.0375**	0.0055	0.0005
**F**	0.0030	0.0220	0.0180	0.0180	0.1870	0.1740	0.1800	0.1870	0.4130	0.4370	0.4430	0.4090
**Lc**	0.5324	0.4352	0.2984	0.1552	0.5580	0.4074	0.3342	0.1772	0.5898	0.4208	0.3414	0.2188
**Ls**	0.6580	0.6090	0.5860	0.5400	0.5890	0.4120	0.3350	0.2980	0.3640	0.1170	**0.0650**	**0.0630**

To control empirical *α* around 0.05, simulated critical values for all tests at all four time lengths using all of the three cases of deterministic component have been found and then by using these simulated critical values, again empirical *α* is calculated and these *α*′*s* are displayed in Table 3. It is evident from Table 3 that all tests have empirical *α* around 0.05 now. It is very important to control empirical *α* around 0.05 in a statistical hypothesis testing, because a large *α* will reduce BETA, the probability of Type II Error. So, as compared to the considered nominal significance level, a decrease in empirical *α* leads to an increase in BETA and an increase in empirical *α* leads to a decrease in BETA resulting in a meaningless conclusion.

**Table 3:** Probabilities of type I error "*α*" using simulated critical values.

Tests	D^0^LT^0^				D^1^LT^0^				D^1^LT^1^			
	Time Length *T*				Time Length *T*				Time Length *T*			
	30	60	120	240	30	60	120	240	30	60	120	240
**LM**	0.0476	0.0499	0.0510	0.0478	0.0514	0.0538	0.0482	0.0479	0.0454	0.0467	0.0468	0.0487
**H1**	0.0523	0.0502	0.0491	0.0443	0.0506	0.0481	0.0502	0.0501	0.0480	0.0507	0.0511	0.0505
**H2**	0.0509	0.0558	0.0484	0.0485	0.0518	0.0513	0.0442	0.0490	0.0561	0.0512	0.0472	0.0480
**Sc**	0.0509	0.0529	0.0519	0.0473	0.0485	0.0548	0.0521	0.0501	0.0542	0.0498	0.0485	0.0481
**LBI**	0.0489	0.0505	0.0498	0.0511	0.0490	0.0513	0.0497	0.0513	0.0416	0.0517	0.0452	0.0473
**F**	0.0467	0.0441	0.0525	0.0472	0.0390	0.0517	0.0542	0.0482	0.0536	0.0542	0.0524	0.0459
**Lc**	0.0442	0.0478	0.0492	0.0500	0.0484	0.0492	0.0496	0.0490	0.0478	0.0500	0.0534	0.0540
**Ls**	0.0370	0.0430	0.0450	0.0640	0.0470	0.0460	0.0630	0.0380	0.0380	0.0530	0.0570	0.0570

As the use of simulated critical values led to controlled empirical *α* around 0.05, the significance level considered in this paper. So, these simulated critical values are used to estimate the probabilities of Type II Error i.e. *β*. These *β*′*s* for the three cases of deterministic component (*D*^*0*^*LT*^*0*^, *D*^*1*^*LT*^*0*^ and *D*^*1*^*LT*^*1*^) are displayed in Tables 4–6 respectively. The test having the minimum *β* is marked as "A” and declared as best performer in this study. Similarly, the tests having 2^nd^ minimum and 3^rd^ minimum are marked as "B" and "C" and nominated as better performer and good performer respectively in this study. These nominations are done for each alternative hypothesis i.e. *γ*.

**Table 4:** Probabilities of type II error "*β*" for D^0^LT^0^.

T = 30								
*γ*	LM	H1	H2	Sc	LBI	F	Lc	Ls
**0.9**	0.760^A^	0.939	0.935	0.888 ^C^	0.919	0.887 ^B^	0.947	0.940
**0.8**	0.619 ^A^	0.914	0.901	0.835 ^B^	0.906	0.860 ^C^	0.926	0.922
**0.7**	0.520 ^A^	0.876	0.856	0.815 ^B^	0.898	0.846 ^C^	0.923	0.918
**0.6**	0.433 ^A^	0.838	0.807 ^C^	0.792 ^B^	0.889	0.840	0.908	0.915
**0.5**	0.383 ^A^	0.791	0.751 ^B^	0.783 ^C^	0.885	0.838	0.901	0.909
**0.4**	0.341 ^A^	0.768 ^C^	0.706 ^B^	0.778	0.884	0.837	0.902	0.901
**0.3**	0.304 ^A^	0.721 ^C^	0.658 ^B^	0.771	0.882	0.837	0.901	0.895
**0.2**	0.284 ^A^	0.701 ^C^	0.626 ^B^	0.775	0.880	0.836	0.900	0.881
**0.1**	0.255 ^A^	0.675 ^C^	0.598 ^B^	0.768	0.878	0.835	0.900	0.875
**0.0**	0.250 ^A^	0.658 ^C^	0.576 ^B^	0.768	0.878	0.834	0.899	0.872
***T* = 60**								
**0.9**	0.613 ^A^	0.915	0.916	0.716 ^B^	0.774 ^C^	0.814	0.856	0.887
**0.8**	0.412 ^A^	0.843	0.832	0.627 ^B^	0.724 ^C^	0.757	0.785	0.876
**0.7**	0.271 ^A^	0.747	0.741	0.589 ^B^	0.696 ^C^	0.749	0.740	0.870
**0.6**	0.210 ^A^	0.666	0.641 ^C^	0.561 ^B^	0.686	0.737	0.719	0.850
**0.5**	0.167 ^A^	0.581	0.560 ^B^	0.564 ^C^	0.683	0.726	0.697	0.842
**0.4**	0.141 ^A^	0.531 ^C^	0.494 ^B^	0.571	0.682	0.724	0.695	0.834
**0.3**	0.116 ^A^	0.490 ^C^	0.438 ^B^	0.554	0.681	0.719	0.693	0.820
**0.2**	0.105 ^A^	0.449 ^C^	0.396 ^B^	0.559	0.676	0.718	0.691	0.819
**0.1**	0.098 ^A^	0.423 ^C^	0.360 ^B^	0.561	0.676	0.715	0.690	0.811
**0.0**	0.093 ^A^	0.398 ^C^	0.338 ^B^	0.560	0.672	0.711	0.687	0.807
***T* = 120**								
**0.9**	0.380 ^A^	0.896	0.884	0.510 ^B^	0.586 ^C^	0.690	0.636	0.890
**0.8**	0.165 ^A^	0.765	0.746	0.396 ^B^	0.532	0.636	0.522 ^C^	0.889
**0.7**	0.085 ^A^	0.642	0.609	0.352 ^B^	0.513	0.633	0.472 ^C^	0.888
**0.6**	0.053 ^A^	0.524	0.495	0.324 ^B^	0.501	0.629	0.466 ^C^	0.879
**0.5**	0.033 ^A^	0.453	0.421 ^C^	0.330 ^B^	0.507	0.619	0.457	0.876
**0.4**	0.028 ^A^	0.374	0.352 ^C^	0.325 ^B^	0.500	0.617	0.463	0.873
**0.3**	0.025 ^A^	0.349	0.301 ^B^	0.325 ^C^	0.494	0.615	0.451	0.869
**0.2**	0.022 ^A^	0.327	0.269 ^B^	0.320 ^C^	0.497	0.614	0.430	0.849
**0.1**	0.019 ^A^	0.296 ^C^	0.243 ^B^	0.323	0.489	0.612	0.425	0.778
**0.0**	0.016 ^A^	0.285 ^C^	0.227 ^B^	0.311	0.500	0.609	0.421	0.683
***T* = 240**								
**0.9**	0.162 ^A^	0.838	0.832	0.284 ^B^	0.376	0.591	0.358 ^C^	0.870
**0.8**	0.042 ^A^	0.644	0.636	0.170 ^B^	0.302	0.553	0.285 ^C^	0.864
**0.7**	0.014 ^A^	0.498	0.471	0.142 ^B^	0.292	0.538	0.252 ^C^	0.862
**0.6**	0.010 ^A^	0.392	0.367	0.140 ^B^	0.287	0.536	0.248 ^C^	0.853
**0.5**	0.005 ^A^	`0.329	0.303	0.139 ^B^	0.277	0.535	0.234 ^C^	0.839
**0.4**	0.003 ^A^	0.275	0.248	0.132 ^B^	0.272	0.535	0.234 ^C^	0.834
**0.3**	0.003 ^A^	0.241	0.210 ^C^	0.132 ^B^	0.279	0.531	0.232	0.829
**0.2**	0.003 ^A^	0.223	0.188 ^C^	0.134 ^B^	0.278	0.529	0.231	0.762
**0.1**	0.002 ^A^	0.210	0.161 ^C^	0.132 ^B^	0.280	0.524	0.231	0.697
**0.0**	0.002 ^A^	0.199	0.146 ^C^	0.131 ^B^	0.270	0.521	0.224	0.514

^A, B^ and ^C^ denote the Best, Better and good performer respectively.

**Table 5:** Probabilities of type II error "*β*" for D^1^LT^0^.

T = 30								
*γ*	LM	H1	H2	Sc	LBI	F	Lc	Ls
**0.9**	0.880^A^	0.945 ^C^	0.949	0.936 ^B^	0.954	0.957	0.945 ^C^	0.950
**0.8**	0.737 ^A^	0.933	0.936	0.906 ^B^	0.948	0.953	0.945	0.909 ^C^
**0.7**	0.596 ^A^	0.914	0.910	0.887 ^B^	0.947	0.951	0.945	0.907 ^C^
**0.6**	0.467 ^A^	0.892	0.882	0.854 ^B^	0.944	0.951	0.944	0.858 ^C^
**0.5**	0.383 ^A^	0.871	0.839 ^C^	0.835 ^B^	0.943	0.953	0.944	0.845
**0.4**	0.313 ^A^	0.842	0.809 ^B^	0.826 ^C^	0.942	0.952	0.943	0.837
**0.3**	0.267 ^A^	0.825	0.764 ^B^	0.820 ^C^	0.943	0.951	0.941	0.829
**0.2**	0.231 ^A^	0.791 ^C^	0.737 ^B^	0.811	0.942	0.950	0.940	0.823
**0.1**	0.195 ^A^	0.786 ^C^	0.696 ^B^	0.804	0.940	0.955	0.938	0.815
**0.0**	0.187 ^A^	0.763 ^C^	0.669 ^B^	0.804	0.939	0.952	0.938	0.807
***T* = 60**								
**0.9**	0.739 ^A^	0.943	0.929	0.822 ^B^	0.887	0.949	0.935	0.861 ^C^
**0.8**	0.461 ^A^	0.900	0.890	0.692 ^B^	0.831	0.945	0.915	0.745 ^C^
**0.7**	0.282 ^A^	0.831	0.818	0.609 ^B^	0.804	0.940	0.904	0.681 ^C^
**0.6**	0.174 ^A^	0.753	0.734	0.576 ^B^	0.788	0.937	0.890	0.667 ^C^
**0.5**	0.117 ^A^	0.699	0.652	0.542 ^B^	0.784	0.938	0.885	0.650 ^C^
**0.4**	0.086 ^A^	0.633	0.568 ^C^	0.534 ^B^	0.787	0.941	0.880	0.647
**0.3**	0.069 ^A^	0.589	0.511 ^B^	0.526 ^C^	0.778	0.940	0.880	0.641
**0.2**	0.055 ^A^	0.542	0.456 ^B^	0.530 ^C^	0.777	0.937	0.876	0.621
**0.1**	0.047 ^A^	0.514 ^C^	0.405 ^B^	0.523	0.776	0.942	0.873	0.619
**0.0**	0.040 ^A^	0.488 ^C^	0.389 ^B^	0.513	0.775	0.943	0.870	0.610
***T* = 120**								
**0.9**	0.461 ^A^	0.924	0.924	0.584 ^B^	0.652	0.936	0.870	0.607 ^C^
**0.8**	0.168 ^A^	0.835	0.833	0.373 ^B^	0.528 ^C^	0.924	0.788	0.576
**0.7**	0.069 ^A^	0.732	0.714	0.303 ^B^	0.498 ^C^	0.920	0.762	0.575
**0.6**	0.032 ^A^	0.625	0.608	0.262 ^B^	0.485 ^C^	0.916	0.742	0.573
**0.5**	0.014 ^A^	0.543	0.512	0.243 ^B^	0.476 ^C^	0.920	0.728	0.536
**0.4**	0.011 ^A^	0.473	0.428 ^C^	0.242 ^B^	0.472	0.917	0.723	0.527
**0.3**	0.007 ^A^	0.434	0.380 ^C^	0.228 ^B^	0.475	0.914	0.721	0.518
**0.2**	0.005 ^A^	0.389	0.336 ^C^	0.227	0.473	0.911	0.714	0.518
**0.1**	0.005 ^A^	0.364	0.297 ^C^	0.225 ^B^	0.472	0.914	0.712	0.513
**0.0**	0.004 ^A^	0.353	0.273 ^C^	0.221 ^B^	0.471	0.914	0.710	0.511
***T* = 240**								
**0.9**	0.156 ^A^	0.883	0.886	0.254 ^B^	0.337 ^C^	0.905	0.674	0.553
**0.8**	0.024 ^A^	0.738	0.716	0.114 ^B^	0.228 ^C^	0.880	0.552	0.544
**0.7**	0.005 ^A^	0.591	0.579	0.083 ^B^	0.196 ^C^	0.865	0.515	0.540
**0.6**	0.002 ^A^	0.477	0.451	0.069 ^B^	0.194 ^C^	0.862	0.497	0.533
**0.5**	0.001 ^A^	0.402	0.359	0.066 ^B^	0.192 ^C^	0.860	0.484	0.517
**0.4**	0.000 ^A^	0.345	0.301	0.061 ^B^	0.189 ^C^	0.849	0.481	0.516
**0.3**	0.000 ^A^	0.292	0.263	0.062 ^B^	0.190 ^C^	0.859	0.474	0.509
**0.2**	0.000 ^A^	0.278	0.228	0.061 ^B^	0.192 ^C^	0.855	0.471	0.453
**0.1**	0.000 ^A^	0.260	0.204	0.061 ^B^	0.189 ^C^	0.859	0.470	0.428
**0.0**	0.000 ^A^	0.241	0.184	0.059 ^B^	0.186 ^C^	0.853	0.469	0.416

^A, B^ and ^C^ denote the Best, Better and good performer respectively.

**Table 6:** Probabilities of type II error "*β*" for D^1^LT^1^.

T = 30								
*γ*	LM	H1	H2	Sc	LBI	F	Lc	Ls
**0.9**	0.926^A^	0.948 ^C^	0.942 ^B^	0.950	0.953	0.946	0.950	0.962
**0.8**	0.864 ^A^	0.943	0.938 ^B^	0.941 ^C^	0.953	0.950	0.955	0.945
**0.7**	0.733 ^A^	0.935 ^C^	0.932 ^B^	0.939	0.949	0.953	0.947	0.967
**0.6**	0.606 ^A^	0.926 ^B^	0.929	0.930	0.949	0.959	0.945	0.968
**0.5**	0.485 ^A^	0.921 ^C^	0.919 ^B^	0.929	0.943	0.960	0.952	0.968
**0.4**	0.377 ^A^	0.920 ^C^	0.913 ^B^	0.918	0.941	0.963	0.944	0.962
**0.3**	0.299 ^A^	0.908 ^C^	0.906 ^B^	0.909	0.940	0.963	0.944	0.956
**0.2**	0.246 ^A^	0.908 ^C^	0.892 ^B^	0.908 ^C^	0.939	0.966	0.952	0.958
**0.1**	0.202 ^A^	0.905	0.886 ^B^	0.899 ^C^	0.935	0.967	0.946	0.951
**0.0**	0.161 ^A^	0.904	0.878 ^B^	0.903 ^C^	0.934	0.968	0.948	0.963
***T* = 60**								
**0.9**	0.855 ^A^	0.949	0.949	0.908 ^B^	0.951	0.952	0.951	0.923 ^C^
**0.8**	0.612 ^A^	0.933	0.932	0.803 ^B^	0.954	0.955	0.956	0.852 ^C^
**0.7**	0.381 ^A^	0.918	0.913	0.707 ^B^	0.943	0.954	0.957	0.829 ^C^
**0.6**	0.229 ^A^	0.894	0.892	0.640 ^B^	0.947	0.958	0.954	0.800 ^C^
**0.5**	0.140 ^A^	0.863	0.852	0.602 ^B^	0.949	0.959	0.956	0.769 ^C^
**0.4**	0.083 ^A^	0.833	0.813	0.575 ^B^	0.949	0.962	0.953	0.765 ^C^
**0.3**	0.052 ^A^	0.814	0.775	0.570 ^B^	0.950	0.961	0.956	0.722 ^C^
**0.2**	0.041 ^A^	0.783	0.732	0.556 ^B^	0.950	0.962	0.955	0.713 ^C^
**0.1**	0.029 ^A^	0.759	0.696 ^C^	0.553 ^B^	0.950	0.959	0.956	0.696 ^C^
**0.0**	0.022 ^A^	0.735	0.657 ^C^	0.554 ^B^	0.950	0.965	0.957	0.692
***T* = 120**								
**0.9**	0.605 ^A^	0.941	0.942	0.724 ^C^	0.826	0.951	0.945	0.700 ^B^
**0.8**	0.227 ^A^	0.916	0.916	0.454 ^B^	0.691	0.946	0.941	0.532 ^C^
**0.7**	0.079 ^A^	0.877	0.865	0.313 ^B^	0.644	0.947	0.929	0.507 ^C^
**0.6**	0.027 ^A^	0.829	0.813	0.263 ^B^	0.622	0.945	0.926	0.486 ^C^
**0.5**	0.012 ^A^	0.774	0.751	0.227 ^B^	0.604	0.946	0.924	0.465 ^C^
**0.4**	0.005 ^A^	0.728	0.685	0.217 ^B^	0.597	0.945	0.921	0.452 ^C^
**0.3**	0.002 ^A^	0.679	0.638	0.205 ^B^	0.593	0.943	0.919	0.417 ^C^
**0.2**	0.002 ^A^	0.634	0.575	0.199 ^B^	0.587	0.945	0.913	0.410 ^C^
**0.1**	0.001 ^A^	0.611	0.536	0.203 ^B^	0.582	0.945	0.911	0.404 ^C^
**0.0**	0.001 ^A^	0.579	0.503	0.198 ^B^	0.589	0.943	0.909	0.402 ^C^
***T* = 240**								
**0.9**	0.231 ^A^	0.934	0.938	0.340 ^B^	0.425 ^C^	0.940	0.898	0.435
**0.8**	0.024 ^A^	0.880	0.883	0.115 ^B^	0.248 ^C^	0.930	0.840	0.416
**0.7**	0.004 ^A^	0.806	0.812	0.062 ^B^	0.197 ^C^	0.927	0.824	0.412
**0.6**	0.001 ^A^	0.732	0.714	0.050 ^B^	0.183 ^C^	0.923	0.804	0.408
**0.5**	0.000 ^A^	0.650	0.631	0.039 ^B^	0.170 ^C^	0.924	0.803	0.401
**0.4**	0.000 ^A^	0.585	0.555	0.034 ^B^	0.170 ^C^	0.927	0.805	0.385
**0.3**	0.000 ^A^	0.532	0.493	0.036 ^B^	0.166 ^C^	0.920	0.806	0.381
**0.2**	0.000 ^A^	0.500	0.443	0.030 ^B^	0.162 ^C^	0.924	0.800	0.377
**0.1**	0.000 ^A^	0.463	0.400	0.030 ^B^	0.160 ^C^	0.930	0.793	0.369
**0.0**	0.000 ^A^	0.442	0.369	0.030 ^B^	0.160 ^C^	0.923	0.803	0.343

^A, B^ and ^C^ denote the Best, Better and good performer respectively.

It is evident from Table 4 that for the first case of deterministic part i.e. *D*^*0*^*LT*^*0*^, the LM test is the best performer at all ten *γ*′*s* considered for all four sample sizes. At *T* = 30, for some *γ*′*s*, Sc is better and for some *γ*′*s*, H2 is better performer. Similarly, at the same sample size three tests i.e. H1, H2 and F are good performers at different *γ*′*s*. However, a deep insight of Table 4 reveals that excluding LM, the rest of seven tests have *β*′*s* way larger than 0.5. Moreover, the LM test has also *β*′*s* larger than 0.5 for *γ*′*s* from 0.9 up to 0.7. At a moderate sample size of 60, *β*′*s* tend to improve generally as now *β*′*s* are lesser as compared to the ones for *T* = 30. Now, for majority of *γ*′*s*, H2 is better and H1 is good performer. Again, these two tests (H1 and H2) have *β*′*s* larger than 0.5 for *γ*′*s* from 0.9 to 0.4. The LM test has *β*′*s* larger than 0.5 for only *γ* = 0.9, for rest of *γ*′*s*, *β*′*s* tends to decrease very sharply. When a moderately large sample size of 120 is considered, then *β*′*s* are generally lesser than those for *T* = 30 and *T* = 60. Now Sc is a better performer for majority of *γ*′*s* and H2 is also better performer for some *γ*′*s*. Lc, Sc, H2 and H1 are good performers at various *γ*′*s*. However, LM improves its performance enormously as now it has all *β*′*s* lesser than 0.1 except for two *γ*′*s* i.e. *φ* = 0.9, 0.8. For the large time length of 240, all tests tend to improve their performance as compared to previous three time lengths i.e. *T* = 30, 60, 120. At *T* = 240, LM is the best performer and it has *β*′*s* lesser than 0.05 for all *γ*′*s* except *γ* = 0.9. Sc is better and Lc and H2 are good performers.

For the second case of deterministic component i.e. *D*^*1*^*LT*^*0*^, again LM has the least *β*′*s* at all *γ*′*s* for all *T*. Table 5 depicts that at *T* = 30, the smallest sample size considered in the study, LM is the best, Sc and H2 are better and Sc, H1 and Ls are good performers. However, all these tests have *β*′*s* way greater than 0.5 except LM at some *γ*′*s*. At *T* = 60, the same performance of *T* = 30 is repeated, however, now all tests have *β*′*s* lesser than those for *T* = 30. Especially LM has all *β*′*s* lesser than 0.5 except *γ* = 0.9. At a moderately large *T* = 120, all the tests have improved their performance by having lesser *β*′*s* as compared to *T* = 30 *and* 60. For *T* = 120, LM remains the best, however, Sc becomes better and LBI and H2 becomes good performers. At this *T* = 120 LM has all *β*′*s* lesser than 0.05 for all *γ*′*s* except *γ* = 0.9, 0.8, 0.7. Similarly, Sc has all *β*′*s* lesser than 0.5 for all *γ*′*s* except *γ* = 0.9. At the largest *T* = 240, almost same performance of *T* = 120 is repeated with only one addition that now LBI is the sole good performer. At *T* = 240 these three tests LM, Sc and LBI have *β*′*s* lesser than 0.4.

For the final case of deterministic part i.e. *D*^*1*^*LT*^*1*^ considered in this study, again LM is the sole best performer at all *γ*′*s* for all *T*. However, a detailed inspection of Table 6 reveals that at smallest *T* = 30, all eight tests have *β*′*s* way larger than 0.5 except LM at some *γ*′*s*. H2 is better and H1 is good performer at *T* = 30. At *T* = 60, again same is the case that all tests have *β*′*s* larger than 0.5 except LM at *γ* = 0.9 *and* 0.8. However, now Sc is better, and Ls is good performer. When a moderately large *T* = 120 is considered then in general all tests and especially three tests i.e. LM, Sc and Ls improve their performance as these tests have *β*′*s* lesser than 0.5 for most of *γ*′*s*. Now LM has *β*′*s* lesser than 0.1 for all *γ*′*s* except *γ* = 0.9 *and* 0.8. The better and good performers are Sc and Ls respectively at *T* = 120. At largest sample size of *T* = 240 considered in this study, LM, Sc and LBI are best, better and good performers respectively. However, now LM has *β*′*s* lesser than 0.05 for all *φ*′*s* except *γ* = 0.9. Similarly, Sc has *β*′*s* lesser than 0.05 for all *φ*′*s* except *γ* = 0.9, 0.8 *and* 0.7.

## 4. Conclusions and recommendations

This study is aimed to evaluate the performance of null of cointegration tests on basis of probabilities of Type I and II Errors using Monte Carlo Simulations. In light of discussion in Section 3 it is concluded that use of asymptotic critical values for these eight tests considered in this study, led to distortion in probabilities of Type I Error. At almost all of specifications of deterministic part and sample sizes, these eight tests had probabilities of Type I Error way greater or way smaller than the nominal significance level considered. This means that these eight tests faced problems of over rejection as well as under rejection. In statistical and econometric analysis these two problems of over rejection and under rejection have worse implications, leading to useless conclusions and recommendations. As stated by [34], the claims about the robustness of type I error probabilities are false if the asymptotic critical values are used.

To solve these problems of over rejection and under rejection, simulated critical values were estimated and then again probabilities of Type I Error were calculated. The use of simulated critical values led to the control of probabilities of Type I Error around nominal significance level. For further evaluation of performance on basis of probabilities of Type II Error, these simulated critical values were used to ensure that the probabilities of Type I Error are around nominal significance level of 0.05 for all eight tests.

The LM test was the sole best performer at all alternative hypotheses for all specifications of deterministic part and sample sizes on basis of probability of Type II Error. It had probabilities of Type II Error way lesser than its competitors. However, at small time lengths of 30 and 60, even it was the best performer, it had probabilities of Type II Error larger than 0.5. The LM test is a better performer as it uses the contemporaneous variance estimator and the rest used different form of long run variances. Therefore the performances of the rest of the tests also depend upon the selection of type of long run variance and their weighting function. However, the LM test is free of these decisions. Moreover, its underlying assumptions and theory meets with the data generating process used in the current study. From the rest of tests, two tests had better and good performance over all and these tests are Sc and H2 developed. At large sample sizes of 120 and 240, LM test had the extra ordinary least probabilities of Type II Error, even lesser than 0.05. The rest of five tests excluding LM, Sc and H2, performed badly even worst with one exception that LBI performed better for *D*^*1*^*LT*^*0*^ and *D*^*1*^*LT*^*1*^ at time length of 240.

In the light of conclusions, it is recommended that use of asymptotic critical values may be avoided. Furthermore, the use of simulated critical values is highly recommended. In modern age, the use of simulated critical values is easily possible due to the availability of fast computers. While testing for possible cointegration using null of cointegration tests, the LM test may be given priority as compared to the others. For large time lengths of 240 or more, when the presence of only intercept is assumed then LBI test may also be used.

After conducting the current study, few gaps in the literature have been observed which can be pursued in the future starting with the development of a new test of null cointegration on basis of vector autoregressive model as there is no such kind of test available in the literature. Moreover, it can also be assessed that how these current tests are performing when the data are generated using a multivariate system having more than one cointegrating vector.

## Supporting information

S1 File MATLAB codes. (ZIP)
